# A network-based model to explore the role of testing in the epidemiological control of the COVID-19 pandemic

**DOI:** 10.1186/s12879-020-05750-9

**Published:** 2021-01-12

**Authors:** Yapeng Cui, Shunjiang Ni, Shifei Shen

**Affiliations:** 1 Institute of Public Safety Research, Tsinghua University, Beijing, China; 2 Department of Engineering Physics, Tsinghua University, Beijing, China; 3 Beijing Key Laboratory of City Integrated Emergency Response Science, Beijing, China

**Keywords:** Testing, COVID-19, Infectious disease control, Complex networks, Numerical simulation

## Abstract

**Background:**

Testing is one of the most effective means to manage the COVID-19 pandemic. However, there is an upper bound on daily testing volume because of limited healthcare staff and working hours, as well as different testing methods, such as random testing and contact-tracking testing. In this study, a network-based epidemic transmission model combined with a testing mechanism was proposed to study the role of testing in epidemic control. The aim of this study was to determine how testing affects the spread of epidemics and the daily testing volume needed to control infectious diseases.

**Methods:**

We simulated the epidemic spread process on complex networks and introduced testing preferences to describe different testing strategies. Different networks were generated to represent social contact between individuals. An extended susceptible-exposed-infected-recovered (SEIR) epidemic model was adopted to simulate the spread of epidemics in these networks. The model establishes a testing preference of between 0 and 1; the larger the testing preference, the higher the testing priority for people in close contact with confirmed cases.

**Results:**

The numerical simulations revealed that the higher the priority for testing individuals in close contact with confirmed cases, the smaller the infection scale. In addition, the infection peak decreased with an increase in daily testing volume and increased as the testing start time was delayed. We also discovered that when testing and other measures were adopted, the daily testing volume required to keep the infection scale below 5% was reduced by more than 40% even if other measures only reduced individuals’ infection probability by 10%. The proposed model was validated using COVID-19 testing data.

**Conclusions:**

Although testing could effectively inhibit the spread of infectious diseases and epidemics, our results indicated that it requires a huge daily testing volume. Thus, it is highly recommended that testing be adopted in combination with measures such as wearing masks and social distancing to better manage infectious diseases. Our research contributes to understanding the role of testing in epidemic control and provides useful suggestions for the government and individuals in responding to epidemics.

## Background

According to statistics from the World Health Organization (WHO), as of August 28, 2020, there have been over 24 million confirmed cases of coronavirus disease (COVID-19) and over 820,000 related deaths worldwide [1]. The International Monetary Fund (IMF) predicted that the global economic growth would reach -4.9% in 2020 as a result of the COVID-19 pandemic [2]. In order to reduce losses caused by COVID-19, testing has been adopted by many countries as an effective response measure. The WHO has also called for more tests in response to COVID-19 [3]. Researchers have found that testing plays an important role in controlling the spread of infectious diseases [4–9]. Testing can identify individuals who are infected but remain undiagnosed, which makes it possible to protect others from infection by quarantining those who are infected [10–13]. Scholars have also found that testing data can provide accurate estimates of epidemic trends and help governments distinguish whether an outbreak is increasing or past its peak [14]. Testing is so important for controlling epidemics that it has increasingly attracted the attention of scholars.

A subset of previous research on testing focused on trials and clinical statistics, mainly in the field of HIV. In the HIV Prevention Trials Network (HPTN) 071 community-randomized trial [15], participants were divided into three groups: a combination of prevention intervention with universal testing and antiretroviral therapy (ART), prevention intervention with ART provided according to local guidelines, or standard care. The HIV incidence of the three groups showed that universal testing and treatment could reduce the population-level incidence of HIV infection. However, the timing of testing was also found to be important for controlling HIV[16]. Grinsztejn et al. studied the effects of early versus delayed testing on HIV infection, and the clinical results showed that early testing could reduce HIV transmission [13]. Cohen et al. also showed that early testing and implementation of ART treatment could reduce HIV infections [12]. That said, research also showed that the effectiveness of testing could be greatly reduced when high-frequency transmitters were not tested or linkage to care was inadequate [17, 18]. In addition, some scholars demonstrated concern about the effectiveness of testing strategies. For example, Lightfoot et al. reported that using a social network strategy to distribute HIV self-test kits could reduce undiagnosed infections [19]. This suggested that factors such as age, residence, and education level should also be taken into consideration to develop more targeted promotion testing strategies [20, 21].

Another subset of previous research explored the impact of testing on epidemic transmission by mathematical models. A series of established mathematical models showed that universal testing could control the epidemic [22–26]. Ng constructed an agented-based model to explore the effect of testing on the COVID-19 epidemic in the United States. They found that broadening testing would accelerate the return to normal life and random testing was too inefficient unless a majority of population was infected [27]. Berger et al. found that testing at a higher rate in conjunction with targeted quarantine policies can dampen the economic impact of the coronavirus and reduce infection peak [28]. Granich et al. proposed a mathematical model to simulate the spread of HIV and found that universal voluntary testing and treatment could drive HIV transmission to an elimination phase within 5 years [22]. Similarly, a compartmental model was proposed by Aronna et al. to study the impact of testing, and an explicit expression for the basic reproduction number *R*_0_ in terms of testing rate was obtained. From the expression of *R*_0_, the conclusion was drawn that testing among asymptomatic cases is fundamental to the control of epidemics [29]. Moreover, Kolumbus and Nisan established a susceptible-exposed-infected-recovered(SEIR) model to study the effect of tracking and testing on suppressing epidemic outbreaks. They found that testing could reduce both economic losses and mortality, but required a large testing capacity [30]. According to a report by the Imperial College London, testing healthcare workers(HCWs) and other at-risk groups weekly could reduce their contribution to transmission by 25-33% [3]. Similarly, Priyanka and Verma adopted the susceptible-infected-recovered(SIR) model to compare the effectiveness of testing and lockdown measures and found that testing outperformed lockdowns [31]. Omori et al. reported that the limited testing capacity had a significant influence on the estimation of epidemic growth rate [32]. The effect of specificity and sensitivity of testing has also been studied [33, 34].

A limitation of previous studies is that they primarily examined infectious diseases with a slow transmission process, such as HIV. In other words, the number of infections remain relatively small over a short period. As a result, the upper bound of testing volume does not need to be considered. However, when epidemics such as SARS and COVID-19 occur, infections multiply rapidly in a short time, and a much larger number of individuals need to be diagnosed through testing. In this case, the upper bound of daily testing volume cannot be ignored, and the impact of testing on suppressing epidemic transmission requires in-depth research. In mathematical models, it is often simply assumed that individuals are tested and quarantined with a certain probability. However, in real life, the daily testing volume gradually increases as the understanding of the epidemic deepens, and an individual is typically not tested again for a certain period (such as two incubation periods) after being tested negative, considering the limited testing resources. In order to bridge the gap, an epidemic transmission model combined with a testing mechanism was proposed to study the role of testing in epidemic control. The paper is organized as follows. In “Methods” section, we state the epidemic transmission model and testing mechanism in detail. In “Results” section, a series of numerical simulations are detailed, and the results are described. The discussion is presented in “Discussion” section, and conclusions are stated in “Conclusions” section.

## Methods

We proposed a model to study the impact of testing on epidemic transmission. The model consists of two parts: an epidemic transmission model, and a testing mechanism. The former simulates the epidemic transmission process in the population, and the latter models the testing process implemented by the government. We also stated the strategy used to validate the proposed model.

### Epidemic transmission model

Complex networks have been a good framework for describing the population structure in real world. A network is composed of nodes and edges. Nodes represent individuals and edges represent social contacts between individuals. The number of edges connected with a node is called the degree of the node. Studies have shown that the degree distribution of social networks obeys a power-law distribution [35–37], which indicates that vast majority of individuals have small degrees, but there exist some individuals who contact with many individuals (also called super spreaders in the context of epidemics). These networks whose degree distribution obeys the power-law distribution are called scale-free networks, and the Barabasi-Albert (BA) network [38] is one kind of scale-free networks. When generating the BA network, we start with a small nucleus of *m*_0_ connected nodes. Then, a new node is added every step to connect to the old nodes. The probability of the new node connected to node *i* is proportional to the degree of node *i*. After enough steps, a network with a power-law degree distribution will be generated. Then, we simulate the epidemic transmission process on the generated networks.

**Fig. 1:**
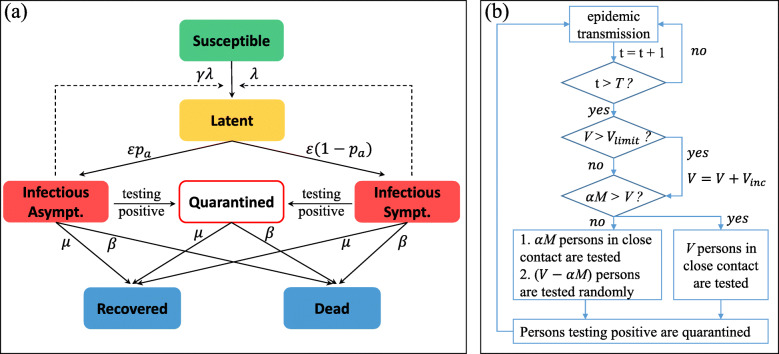
A diagram illustrating the proposed model. **a** shows the epidemic transmission process and (**b**) shows the testing mechanism. The descriptions of parameters in the figure are described in Table 1

In this study, an extended SEIR model [39, 40] was introduced to describe the epidemic transmission process. In our model, an individual can be classified into one of six states: susceptible (S), latent (L), asymptomatic infectious (*I*_*a*_), symptomatic infectious (*I*_*s*_), recovered (R), and dead (D). Specifically, the infection process is as follows. Initially, an individual is randomly chosen as the infection source (*i.e.*, set it in state *I*_*s*_) and others are susceptible (S). At each time step, a susceptible (S) individual *i* randomly contacts one of their neighbors. Individual *i* in contact with symptomatic or asymptomatic infectious individuals will be infected with a probability of *λ* and *γλ*, respectively, where *λ* represents the infection rate in contact with symptomatic infectious individuals, and *γ* measures the relative infectiousness of asymptomatic infections compared with symptomatic infections. Once individual *i* is infected, they will enter the latent (L) state, and at the end of the latency period 1/*ε*, they will become asymptomatic or symptomatic infectious, with the probability *p*_*a*_ and 1−*p*_*a*_, respectively. At the same time, infectious individuals (asymptomatic and symptomatic) will recover with probability *μ* and die at rate *β*. The whole process will continue to evolve until there are no infected individuals (latent, asymptomatic, or symptomatic) on the networks. Figure 1a describes the epidemic transmission process.

**Table 1:** Model parameters, variables and respective descriptions

Parameters	Description	Value
*N*	Number of nodes (population size)	Different values
*λ*	Infection rate in contact with symptomatic individuals	Different values
*γ*	Relative infectiousness of asymptomatic individuals	0.5 [39, 40]
*ε*	Reciprocal of latency period	0.2 [39, 40]
*p*_*a*_	Transmission rate from state *E* to *I*_*a*_	0.4 [39, 40]
*μ*	Recovery rate	0.2 [39, 40]
*β*	Death rate	0.03 [39, 40, 43]
*T*	Testing start time	Different values
*V*	Daily testing volume (normalized by population *N*)	Different values
*α*	Contact-tracking testing preference	Different values
*V*_*inc*_	Increase speed of daily testing volume	Different values
*V*_*limit*_	Limit of daily testing volume	Different values

### Testing mechanism

In real life, we are not typically aware of infectious diseases from the time they occur, and thus there is a delay between the start of testing and the time when infectious diseases begin. Therefore, in our model, only when the current time step is greater than *T* will the testing mechanism be introduced into the epidemic transmission model. In addition, because of limited healthcare workers and medical resources, an upper bound exists in daily testing volume. At each time, the largest number of people who can be tested is *V*, which represents the daily testing volume. In this model, asymptomatic and symptomatic infectious individuals will test positive and will be quarantined, and thus they cannot cause secondary infections by contact with others. Given the limited testing resources, individuals who are tested negative will not be tested again within two incubation periods, which has been adopted by many countries as a testing strategy in response to COVID-19 [41, 42].

As the understanding of the epidemic deepens, the daily testing volume will gradually increase. Considering the limited medical staff and their working hours, there is also an upper bound on the daily testing volume. The change in daily testing volume is

1 $$ V = max (V_{inc}\times(t-T),V_{limit}),  $$

where *V*_*inc*_ and *V*_*limit*_ indicate the increase speed and upper bound of the daily testing volume, respectively; *t* is the current time step; and *T* is the time when testing starts.

In addition, different testing strategies may be used when implementing testing, such as random testing (RT), contact-tracking testing (CT), or a combination of the two. In this study, testing preference, *α*, which measures the priority of testing individuals who are in close contact with confirmed cases, was introduced to represent different testing methods. If *α*=1, individuals in close contact with confirmed cases will be tested first (CT). Moreover, *α*=0 means random testing (RT), and when 0<*α*<1, a combination of RT and CT is adopted.

The testing process is performed as follows. *M* represents the number of individuals who are in close contact with confirmed cases and not tested. At each time step, if *αM*≤*V*, *αM* individuals in close contact will be tested first, then *V*−*αM* individuals will be tested randomly in the population. Otherwise, if *αM*>*V*, only *V* individuals in close contact will be tested randomly. Figure 1b illustrates the testing process. Table 1 presents a summary of parameters and variables, and respective descriptions as well as values used in our model.

### Model validation

We compared the simulation data with real data to validate our model. In response to the COVID-19 pandemic, many countries have adopted testing measures. As a result of the different testing capabilities, the number of people tested every day varies in different countries. In this study, the testing-positive rate was used as an indicator to compare the simulation results with real data. The reason why the number of confirmed cases is not used is that we think the number of confirmed cases refers to the number of infected individuals identified by testing, but there are still many infected individuals who have not been tested in the population. Therefore, it is not appropriate to use the number of confirmed cases to estimate the actual infection scale in the population. Considering that the testing process can be regarded as a sampling of the population, the testing-positive rate can represent the actual infection scale in the population to some extent. Therefore, when verifying the proposed model, we used the peak of the testing-positive rate curve to represent the peak of infection scale in the population.

Specifically, the real data came from the daily report of each country and was collected by Our World in Data [44]. The real data included the number of people who had been tested and the number of people who had tested positive (confirmed cases) every day. Based on the data, the testing-positive rate was calculated. For country *i*, its testing-positive rate curve reaches the peak *P*_*i*_ on date *T*_*i*_. We let $V_{i}^{t}$ be the testing volume of country *i* on date *t*. We calculated the average of $V_{i}^{t}$ where *t*<*T* and obtained the average testing volume of country *i* denoted as *V*_*i*_. The testing volumes after date *T* were not considered because these testing volumes do not contribute to the peak of the positive rate curve. After calculation process described above, we obtained a pair of values (*V*_*i*_, *P*_*i*_) for each country. At the same time, based on our proposed model, we obtained the peak of the testing-positive rate curve under different testing volumes. In the context of COVID-19, we set the basic reproduction number *R*_0_=2.6 [43]. Contact-tracking testing preference *α* was set as 1, indicating that the individuals in close contact with confirmed cases will be tested with high priority; this has been adopted by most countries as their testing strategy. The calculation process for simulation data was the same as that for real data above. We also obtained a pair of values (*v*_*i*_, *p*_*i*_) for each simulation. If (*v*_*i*_, *p*_*i*_) curve is consistent with (*V*_*i*_, *P*_*i*_) curve, the proposed model will be validated.

## Results

In this study, Barabasi-Albert (BA) scale-free networks were generated and used to describe the contact structure of population in real life [38]. A series of epidemic spread simulations were conducted on these networks. All the results were averaged over 1000 simulations.

**Fig. 2:**
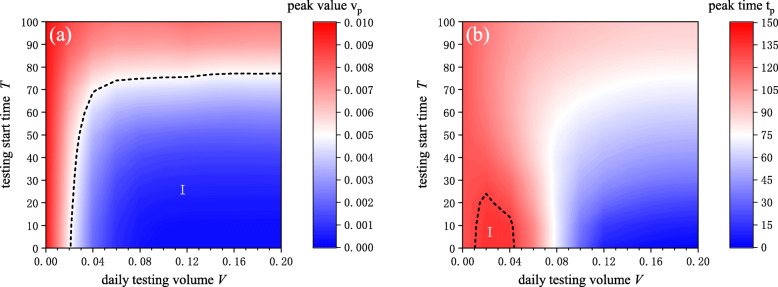
The impact of testing volume *V* and testing start time *T* on epidemic transmission. **a** shows the impact on the infection peak. The red and blue color refer to high and low peak values, respectively. **b** shows the impact on the arrival time of infection peak. The blue color means that the epidemic breaks out very early, while the red color means the opposite. In region I of (**a**), the peak values were smaller than 0.005. In region I of (**b**), the peak times were larger than 130 time steps. Starting testing early and increasing daily testing volume could suppress the epidemic transmission

We first investigated the impact of the daily testing volume and the testing start time on the epidemic transmission. Two indicators were considered: the peak value of infections, *v*_*p*_, and the time when the peak arrives, *t*_*p*_, because these two indicators are of the most concern to governments in their response to epidemics. As Fig. 2a shows, the greater the daily testing volume and the earlier the testing started, the lower the infection peak. To make *v*_*p*_ less than 0.5%, the daily testing volume had to be at least 0.02 and testing had to start within 70 time steps (region I in Fig. 2a). As Fig. 2b shows, *t*_*p*_ first increased and then decreased as the daily testing volume grew. This can be explained as follows. Increasing the daily testing volume can suppress the spread of infectious diseases and delay the outbreak. However, if the testing volume continues to increase, the infectious disease can be controlled to a great extent and will end early because almost all infections are identified and quarantined, leading to a smaller *t*_*p*_. Moreover, *t*_*p*_ reached the maximum when the daily testing volume was between 0.01 and 0.04, and testing started within 25 time steps (region I in Fig. 2b). Further, the larger the *t*_*p*_, the more time there was expected to prepare for the outbreak, which can be very meaningful in controlling epidemics. The findings indicated that the greatest impact of testing on the spread of infectious diseases lies in flattening the infection curve, delaying the arrival of the outbreak, or ending epidemics early. It is recommended that the government start a wide range of tests as soon as possible to suppress epidemic transmission.

**Fig. 3:**
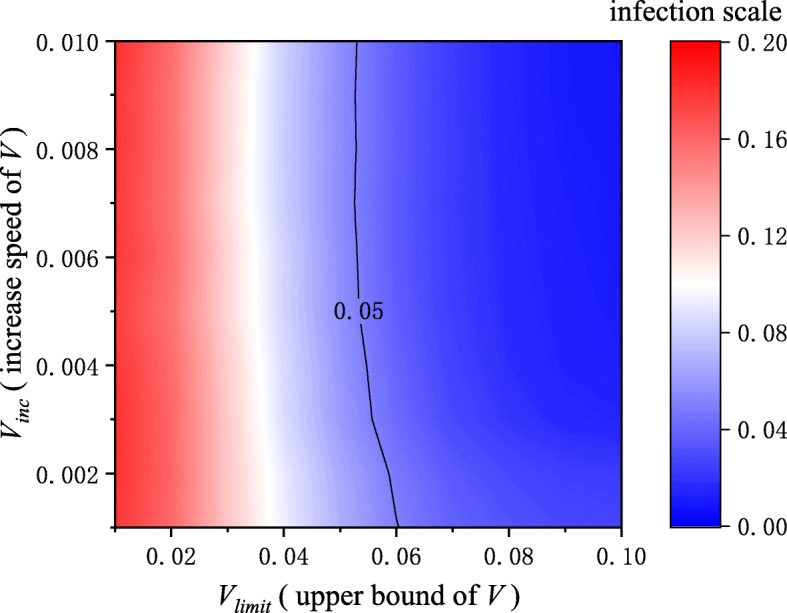
The impact of changes in daily testing volume on infection scale. The red color means the large infection scale, while the blue color means the opposite. Breaking through the limitations of daily testing volume could greatly suppress the epidemic transmission, but promoting the increase speed of daily testing volume hardly changes the infection scale

In real life, the daily testing volume will gradually increase as understanding of the epidemic deepens. Therefore, we studied the impact of changes in the daily testing volume on epidemic transmission. The impact of *V*_*inc*_ and *V*_*limit*_ is shown in Fig. 3. As *V*_*limit*_ increased, the infection scale decreased significantly. However, the infection scale was hardly changed with the increase of *V*_*inc*_, indicating that in terms of controlling infectious diseases, it is more important to break through the limitation of daily testing volume. The solid line in Fig. 3 represents the contour line where the infection scale is 5%, which required the upper bound of daily testing volume to reach at least 5%. The results showed that increasing the upper limit of daily testing volume was essential to control epidemics, which requires the government to invest enough support resources.

**Fig. 4:**
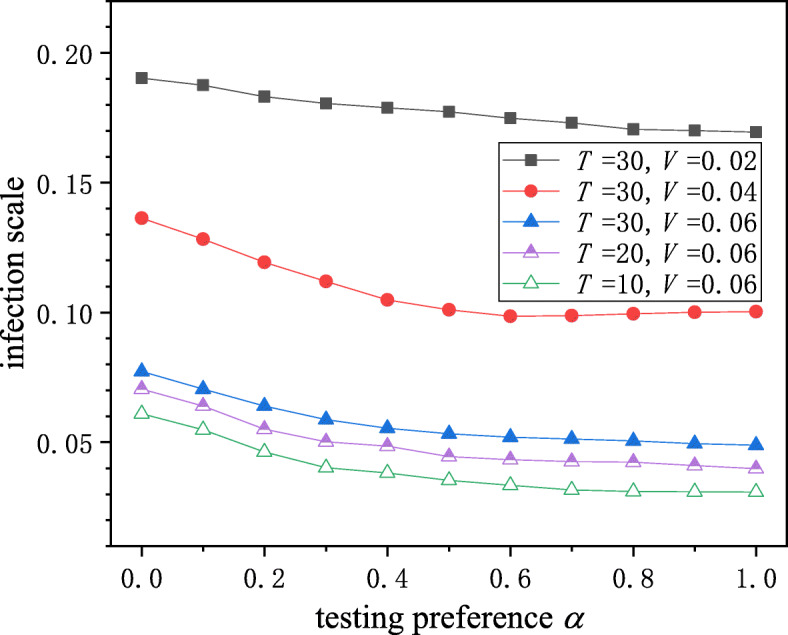
The impact of testing preference on epidemic transmission. Square, circle and triangle curves were obtained under *T*=30 (Group A) and solid, semi-solid and hollow triangle curves were obtained under *V*=0.06 (Group B). Priority testing for individuals in contacts with confirmed cases can suppress the epidemic transmission

We then investigated the impact of testing preference, *α*, on the epidemic transmission, which is shown in Fig. 4. When the testing start time *T* and the daily testing volume *V* were fixed, the larger the *α*, the smaller the final infection scale, which indicated that the higher priority testing is for individuals in contact with confirmed cases, the greater control we can have over infectious diseases. The five curves in Fig. 4 could be divided into two groups according to the values of *T* and *V*: Group A included solid square, solid circle, and solid triangle curves, and Group B included hollow, semi-solid, and solid triangle curves. From Group A and B, we can see that the earlier that testing started and the larger the daily testing volume, the smaller the infection scale. However, comparing Group A and B, it was found that the testing volume *V* had a greater impact on the curve, indicating that the testing volume plays a greater role in controlling the spread of infectious diseases than the testing start time. The findings suggested that the government adopt contact-tracing testing strategy because contact-tracing testing could effectively suppress the spread of infectious diseases.

**Fig. 5:**
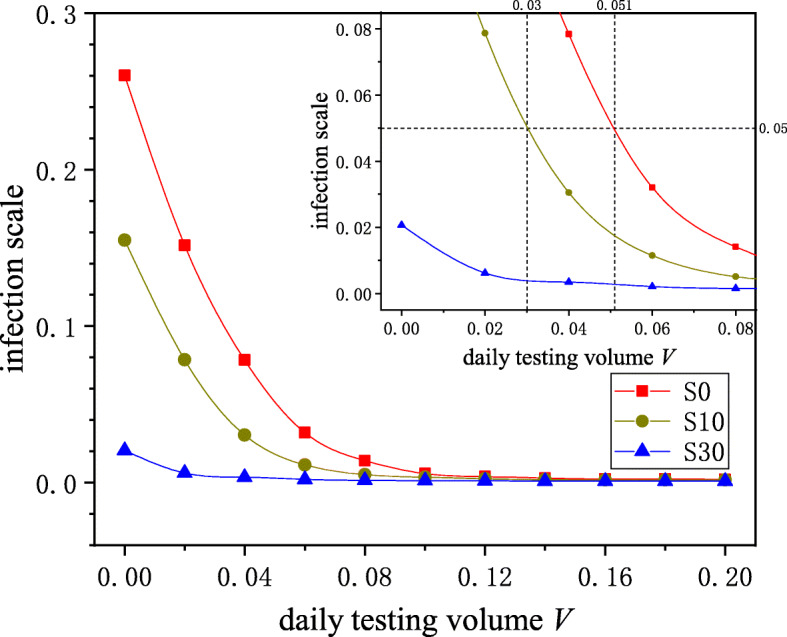
The effect of testing on epidemic transmission under different scenarios. S0 means that no other measures were taken except testing. S10 and S 30 indicate the scenarios where other measures were taken to reduce individuals’ infection probability by 10% and 30%, respectively. Combined with other measures such as wearing masks and social distancing, the daily testing volume could be significantly reduced while the epidemic will still be controlled

In order to control infectious diseases merely through testing (S0), a huge daily testing volume was required (see Fig. 2). Assuming that a city has a population of 10 million, a daily test volume of 5% means that 500,000 individuals need to be tested every day, which is difficult to implement. In order to reduce the testing volume while achieving the goal of controlling infectious diseases, we introduced other control measures such as wearing masks and social distancing. According to reference [45–47], we assumed that social distancing could reduce individuals’ infection probability by 30%(S30). Some scholars revealed that wearing masks had limited effects on epidemic transmission because masks cannot filter submicron-sized airborne particles [45, 48]. However, some studies also showed that wearing masks could prevent the transmission of coronaviruses [49–52]. Considering the debate on the effectiveness of wearing masks, we assumes that wearing masks could reduce the individuals’ infection probability by 10%(S10). As Fig. 5 shows, even if the infection probability was reduced by only 10%, the infection scale was greatly reduced. When the infection probability reduced by 30%, the infection scale was less than 2%. In the inset of Fig. 5, the three different scenarios are compared in detail. To control the infection scale below 5%, if no other measures are taken, the daily testing volume had to reach 5.1%. However, if other measures were taken to reduce the infection probability by 10%, the daily testing volume reduced by more than 40% and only had to reach 3%. Once other measures were taken to reduce the infection probability by 30%, the infection scale was about 1% even if the daily testing volume was 1%. The results indicated that comprehensive measures performed better than single measure. Other measures can greatly reduce the testing volume required to control infectious diseases, relieving the medical resource pressure during epidemic outbreaks.

**Fig. 6:**
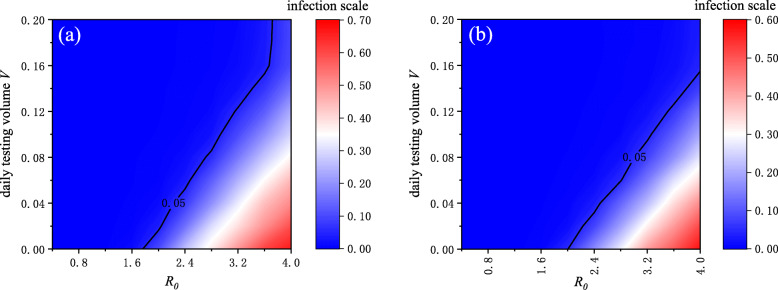
The effect of basic reproductive number *R*_0_ and testing on infection scale under different scenarios. The results of scenario S0 where only testing measure was adopted are shown in (a), and (b) describes the results of scenario S10 where other measures were implemented to reduce individuals’ infection rate by 10%. The solid line is the contour line where the infection scale is 5%. The daily testing volume required to control epidemics increased almost linearly as *R*_0_, but when other measures were adopted, the required testing volume decreased

We further explored how testing affects epidemic transmission when the infectiousness of the epidemic changes. With a different basic reproductive number, *R*_0_, and daily testing volume, *V*, a series of simulations were conducted. The results under scenario S0 and S10 are shown in Fig. 6a and b respectively. S10 means that other measures were adopted to reduce individuals’ infection rate by 10%, and S0 indicates that only testing was adopted. We found that regardless of scenarios S0 and S10, the infection scale always increased with *R*_0_ and decreased with the daily testing volume. The solid line in Fig. 6 is the contour line where the infection scale is 5%, which means the change of minimum daily testing volume required to keep the infection scale below 5%. It can be seen that regardless of whether other measures were taken, the required daily testing volume almost increased linearly as the basic reproductive number grew. However, in scenario S0, when *R*_0_ was relatively large (*R*_0_>3.6), and the required daily testing volume increased sharply (see Fig. 6a), indicating that when the infectiousness of the epidemic is strong, the daily testing volume required to control the epidemic will be extremely large if only the testing measure is taken. Comparing Fig. 6a and b, we also concluded that the required daily testing volume will be greatly reduced if other measures are taken at the same time.

**Fig. 7:**
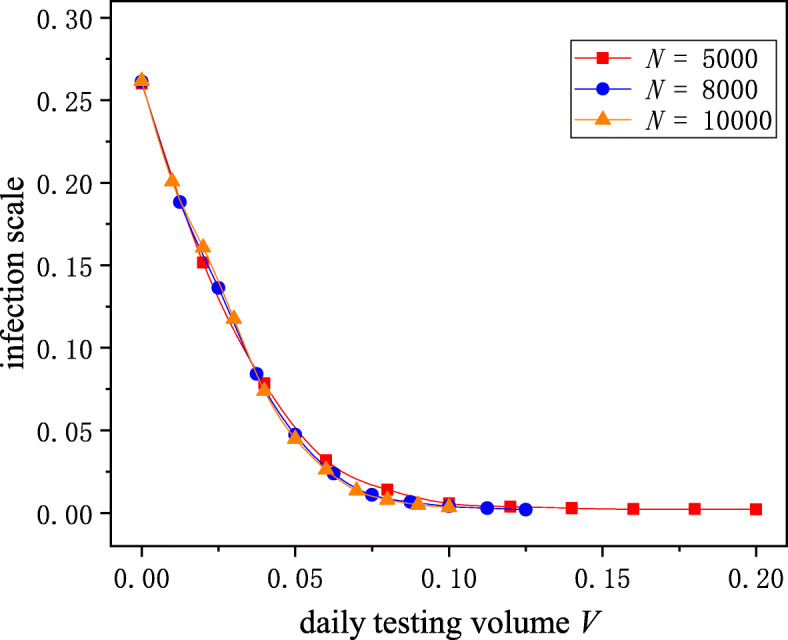
The impact of network scale. The square, circle and triangle curves represent the simulation results on networks with 5000, 8000, and 10000 nodes, respectively. Even if the network scale was different, the trend of the infection scale with the daily testing volume was almost the same

Aiming to study whether the network scale has an impact on the results, we conducted a series of simulations on different networks. As Fig. 7 shows, although the number of nodes in the network is different, the trend of the infection scale with the daily testing volume was almost the same, which indicated that our results are useful for understanding the epidemic transmission process on a larger scale even though they were obtained in a small network.

**Fig. 8:**
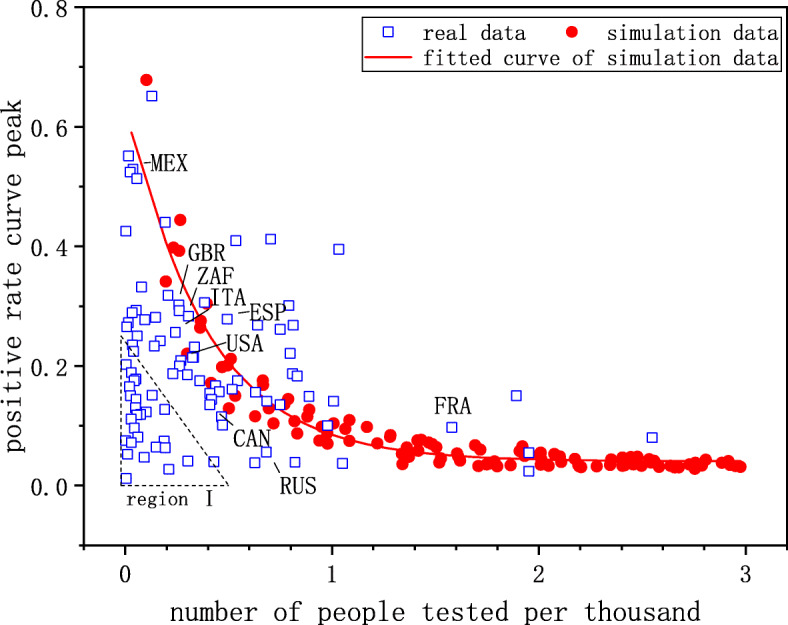
The simulation data versus real data. A hollow square point (real data) indicates one country, representing the average testing volume (x-axis) and the peak of the testing-positive rate curve (y-axis). The red circle points show the peak of positive rate curve under different testing volumes in the simulations. We set *R*_0_=2.6 and *α*=1

Finally, we compared the simulation data with real data to validate our model, as shown in Fig. 8. We fitted the simulation data as shown in the red line. In Fig. 8, ISO country code was used to mark the points of some countries where a large of confirmed cases have been reported, such as the United States, France, Italy, and South Africa. The real data were consistent with the simulation data. Most of the points representing different countries fall near the simulation data. In region I, the errors between real data and simulation data were large. The number of countries falling in region I was 28, which accounts for less than 30% of all countries (95) in the figure. The countries in region I include Bangladesh, Pakistan, the Philippines, Nigeria, Kenya, Myanmar, Thailand, and Morocco. The x-axis values of the points in region I are relatively small (less than 0.5), indicating that a small number of people can be tested every day in these countries. Therefore, because of the small sample size, the actual infection scale in the population will be biased through the testing data, leading to errors between the simulation data and real data in region I. Another explanation is that the true infection rates in these countries are low which explains the low testing-positive rates. In this case, the basic reproduction number *R*_0_ is less than 1 and epidemic outbreak do not occur in these countries. However, in order to ensure that the infectious disease can spread on the networks, we set *R*_0_ as 2.6 in the simulations. To apply our model to these countries, we need to adjust the model parameters. In general, the data we obtained through the proposed model were consistent with the real data, which indicates that the proposed model is reliable, especially for countries reaching pandemic levels of infection.

## Discussion

In response to epidemics, different testing strategies may be adopted by governments, such as random testing, contact-tracking testing, or a combination of the two methods. Moreover, as the understanding of epidemics deepens, the daily testing volume will gradually increase. However, considering the limited medical staff and their working hours, there is an upper bound to the daily testing volume. Therefore, in this study, an epidemic transmission model combined with testing mechanism was proposed to study the role of testing in epidemic control. The combined model incorporates different testing methods as well as an increased speed and upper bound of the daily testing volume.

Through a series of simulations, we found that testing could inhibit the spread of infectious diseases. In addition, the priority testing for individuals in close contact with confirmed cases could enhance the effect of testing on infectious diseases. However, in order to control the epidemic (i.e., control the infection scale below 5%), the daily testing volume had to reach 5.1%. When the urban population is relatively large, 5.1% means a huge amount of testing every day. Our results were consistent with previous studies that concluded that only large-scale testing can effectively control epidemics [3, 30]. Fortunately, effective algorithms such as group testing have been proposed by other scholars [53–55], and these make it possible to greatly increase the daily testing volume.

We also found that when other measures such as wearing masks and social distancing were adopted, the daily testing volume required was greatly reduced. Assuming that other measures could only reduce individuals’ infection probability by 10%, the daily testing volume required were reduced by more than 40%, which further emphasized the importance of taking comprehensive measures in response to epidemics. We conducted simulations on networks with different scales and obtained the same results, which indicates that our results are also meaningful for epidemic control on a large scale.

In this study, we focused on the impact of testing on the spread of infectious diseases. Therefore, the impact of testing specificity was not considered. How an infected individual can affect the spread of infectious diseases after being tested negative is worthy of further study.

## Conclusions

In this study, an epidemic transmission model combined with testing mechanisms was proposed to study the impact of testing volume, testing start time and testing preferences on the spread of infectious diseases. Through extensive numerical simulations, we made the following observations:

1. The infection peak decreased with an increase of daily testing volume. Early testing could also reduce the infection peak. Increasing the upper bound of daily testing volume could greatly reduce the infection scale, but the increased speed of daily testing volume hardly impacted the infection scale.
2. The higher priority there was for testing individuals in close contact with confirmed cases, the smaller the infection scale. However, when the daily testing volume was large, testing preferences had little impact on the infection scale.
3. When testing was combined with other measures are adopted in response to epidemics, the daily testing volume required was reduced by more than 40% even if other measures could only reduce the infection probability by 10%. Plus, the daily testing volume required increased almost linearly with the basic regeneration number *R*_0_.
4. The scale of the network had little effect on the results. Although the nodes of the networks were different, the trend of infection scale with the daily testing volume was basically the same.

The above findings indicated that testing can reduce the infection peak and delay the outbreak of epidemics. This is very important for governments to deal with epidemics because it means that we have more time to prepare medical resources. Testing has become one of the most effective measures to deal with infectious diseases. We also provided some suggestions for dealing with epidemics. It is important to increase the daily testing volume because a larger testing volume means that more infected people can be identified and then treated, thereby reducing the infection scale and saving more lives. However, in response to the COVID-19 pandemic, some countries are not able to implement large-scale testing. In this case, international cooperation is important in increasing the testing volume and controlling the epidemic, especially in underdeveloped countries. Starting testing as early as possible is another way to suppress the epidemic transmission. In addition, comprehensive measures can greatly reduce the daily testing volume required, and therefore it is recommended that testing be combined with measures such as wearing masks and social distancing. Our proposed model was also validated by COVID-19 testing data. In summary, our research contributes to understanding the role of testing in controlling epidemics and provides useful suggestions for governments and individuals in response to infectious diseases.

## Data Availability

The datasets used and/or analysed during the current study are available from the corresponding author on reasonable request.

## Abbreviations

COVID-19: Coronavirus Disease 2019
SEIR model: Susceptible-exposed-infected-recovered model
RT: Random testing
CT: Contact-tracking testing
